# Demyelination and Cognitive Performance in Long COVID Patients with Insomnia and/or Depression

**DOI:** 10.3390/ijms262412141

**Published:** 2025-12-17

**Authors:** Marina Khodanovich, Daria Kamaeva, Anna Usova, Valentina Pashkevich, Marina Moshkina, Victoria Obukhovskaya, Nadezhda Kataeva, Anastasia Levina, Yana Tumentceva, Maria Shadrina, Ariuna Ranzaeva, Svetlana Vasilieva, Evgeny Schastnyy, Anna Naumova, Mikhail Svetlik

**Affiliations:** 1 Laboratory of Neurobiology, Research Institute of Biology and Biophysics, Tomsk State University, 36 Lenina Ave., Tomsk 634050, Russiamimizyana@gmail.com (Y.T.); nav@uw.edu (A.N.); mihasv@gmail.com (M.S.); 2 Cancer Research Institute, Branch of the Tomsk National Research Medical Center of the Russian Academy of Sciences, Kooperativnyi per. 5, Tomsk 634009, Russia; afina.tsk@gmail.com; 3 Department of Fundamental Psychology and Behavioral Medicine, Siberian State Medical University, 2 Moskovskiy Trakt, Tomsk 634050, Russia; 4 Department of Neurology and Neurosurgery, Siberian State Medical University, 2 Moskovskiy Trakt, Tomsk 634050, Russia; 5 Medica Diagnostic and Treatment Center, 86 Sovetskaya Street, Tomsk 634510, Russia; 6 Department of Affective States, Mental Health Research Institute, Tomsk National Research Medical Center of the Russian Academy of Sciences, Tomsk 634014, Russia; 7 Department of Radiology, School of Medicine, University of Washington, 1959 NE Pacific St., Seattle, WA 98195, USA

**Keywords:** long COVID, insomnia, depression, demyelination, white matter, MRI, macromolecular proton fraction, MPF, cognitive performance, blood biomarkers

## Abstract

Insomnia and depression are severe sequelae of COVID-19 and often occur simultaneously. Our study examined associations of insomnia and/or depression with cognitive impairments, white matter changes, and serum biomarkers. In total, 76 long COVID patients and 22 healthy controls were examined using neuropsychiatric (ISI, HADS, and HDRS) and cognitive (MoCA, Stroop, WMT, and TMT) tests, with their blood biomarkers (anti-SARS-CoV-2, BDNF, anti-S100, anti-MBP, and anti-PLP) investigated, and underwent MRI using macromolecular proton fraction (MPF) mapping to quantify myelination. The Insomnia (n = 14), Depression (n = 12), InsDep (comorbid insomnia–depression, n = 13), and PostCovid (long COVID without depression and insomnia, n = 32) groups were identified based on psychiatric/neurological diagnoses and neuropsychiatric assessment. Cognitive performance was most affected in the Insomnia group in the MoCA and CW Stroop tests. The Depression group underperformed in the TMT and W Stroop task; the InsDep group underperformed in the WMT. The Insomnia group showed the greatest demyelination, affecting commissural (CC and tapetum), projection (CR, IC, CST, cerebral peduncles, CP, and ML), and some association pathways (SLF, SFOF), as well as most juxtacortical regions, the thalamus, and the midbrain; these changes correlated with insomnia severity. The Depression and InsDep groups showed smaller but significant overall demyelination correlated with depression severity. The Depression group exhibited the highest MPF decrease in the globus pallidus, putamen, and external capsule, while the InsDep group demonstrated the highest demyelination of the association pathways IFOF, UF, and cingulum. The anti-PLP levels were the highest in the Insomnia group and correlated with both the persistence of insomnia/depression symptoms and demyelination. Demyelination in long COVID is associated with high levels of myelin-specific autoantibodies, but symptoms of insomnia and/or depression are associated with demyelination of a different set of brain structures.

## 1. Introduction

Sleep disorders are common consequences of COVID-19, as shown in a number of studies [1,2,3,4,5,6]. The International COVID-19 Sleep Study (ICOSS) found that COVID-19 is a strong predictor of sleep disorders [3], and insomnia symptoms are observed in 24.1% of short COVID cases and in 60.6% of long COVID cases [4]. A recent systematic review and meta-analysis examined the prevalence of persistent symptoms in COVID-19 survivors 3 years following the pandemic and showed that 20% of individuals were experiencing at least one persistent long COVID symptom [5]. Insomnia was one of the most common symptoms, observed in 11% of patients 3 years post-COVID [5]. A longitudinal study by Zhu et al. [6] showed that the risk of persistent insomnia 3 years after recovery was associated with COVID-19 severity in the acute phase, unlike other long COVID symptoms.

Symptoms of insomnia include difficulty falling or staying asleep, frequent nighttime awakenings, and/or waking up too early in the morning [7]. These sleep problems negatively impact daytime functioning and can lead to reduced quality of life, including fatigue, mood disruption, working disability, cognitive impairments, and drug and alcohol abuse [8,9]. Moreover, insomnia is often accompanied by the development of other psychiatric disorders, such as bipolar disorder, anxiety, and especially major depressive disorder (MDD) [10,11,12,13].

Although considerable effort has been made by researchers, there is still no unified model explaining the pathophysiological causes and mechanisms of insomnia [7,14,15]. There is growing evidence for the important role of white matter (WM) in the pathophysiology of sleep disorders. Animal studies have shown that myelin-related genes are upregulated during sleep, indicating more active myelination [16]. Furthermore, sleep deprivation affects the length of the node of Ranvier and oligodendrocyte proliferation [16]. The vast majority of neuroimaging studies have found alterations in WM integrity and demyelination associated with sleep disorders [17,18,19,20,21,22,23,24,25,26,27,28,29,30,31,32,33,34,35,36,37]. Most of the above studies used diffusion tensor imaging (DTI), which provides an indirect assessment of WM structure rather than myelin density [38].

The present study utilized macromolecular proton fraction (MPF) mapping to quantify brain demyelination. The MPF parameter has been histologically validated in numerous animal studies and has demonstrated a strong correlation with myelin histology [39,40,41,42,43] and high sensitivity to myelin damage (reviewed in [29]). The fast MPF mapping method [44] showed an independence from magnetic field strength [45], insensitivity to fiber orientation [46] and iron deposition [47], high reproducibility [48], and compatibility with routine MRI equipment and original manufacturers’ pulse sequences [49,50,51,52,53].

Our recent studies demonstrated significant, widespread demyelination [50] and cognitive changes [54] in patients with post-COVID depression. Given the close association between MDD and insomnia, we hypothesized that post-COVID patients with insomnia might also exhibit demyelination and impaired cognitive performance. We also aimed to determine which regions of demyelination are more specifically associated with insomnia and which with depression.

Diffusion tensor imaging (DTI) has shown lower insomnia-related fractional anisotropy (FA) in various WM and deep grey matter (GM) regions, including the internal capsule (IC) [23,25,29], corpus callosum (CC) [23,25,30], corona radiata (CR) [23,25], superior longitudinal fasciculus (SLF) [23,30], thalamus [23], frontal subcortical [22] and fronto-limbic [26] tracts, and the thalamus–pars triangularis [31]. Only two DTI studies found increased FA in patients with insomnia [24,28], and one study found no effect of sleep quality [36].

Our knowledge of WM changes associated with specific COVID-19 consequences, such as insomnia and depression, as well as underlying molecular mechanisms, remains limited. Most studies of post-COVID patients show WM abnormalities [55,56,57,58,59,60,61,62] but do not consider specific symptoms and their relationship with demyelination, which may be caused by inflammation and other factors [63,64,65]. Our recent studies demonstrated neurocognitive changes [54] and widespread demyelination in patients with post-COVID depression, and we proposed the inferior fronto-occipital fasciculus (IFOF) as a key tract responsible for depression as a consequence of COVID-19 [50]. The aim of the present study, performed on the same cohort of patients, was to clarify specific WM changes related to insomnia, depression, and comorbid insomnia–depression as the most severe consequences of COVID-19. Additionally, we examined the serum levels of myelin-specific autoantibodies (anti-MBP and anti-PLP), anti-SARS-CoV-2 spike proteins, and CNS-related proteins (BDNF and anti-S100) as underlying mechanisms of demyelination and neuropsychiatric symptoms in long COVID.

## 2. Results

### 2.1. COVID-19-Related Parameters

The main parameters associated with the COVID-19 disease and its consequences are presented in Table 1. Significant differences were found only for the number of symptoms of long COVID: patients from the InsDep group had a higher number of symptoms than those in the PostCovid comparison group. The number of symptoms in the acute phase of COVID-19 did not differ between groups. No differences were found in disease severity, the number of COVID-19 episodes, and time elapsed since the first and last episodes.

### 2.2. Neuropsychological Results

Table 2 and Table 3 demonstrate the results of the psychiatric and cognitive testing of the study participants. The groups were clearly differentiated by the results of tests related to insomnia and depression. The Insomnia and InsDep groups showed significantly higher ISI compared not only to the control and PostCovid groups but also compared to the Depression group. The Depression and InsDep groups differed significantly not only from both the control and PostCovid groups but also from the Insomnia group patients, showing higher HDRS scores and HADS depression index. The InsDep group revealed significantly higher HADS anxiety index compared to all groups, including the Depression and Insomnia groups. The patients of the InsDep group had the highest total scores in both the HDRS and HADS tests.

Long COVID patients showed worse cognitive performance in different tests compared to the controls. Insomnia patients had significantly lower MoCA total scores and CW time in the Stroop task but better immediate recall in WMT and faster processing time in TMT compared to the Depression and Ins-Dep groups. Patients in the Depression group performed more slowly on the TMT and the Stroop word recall task compared to the Control, PC, and Insomnia groups (only for TMT). Patients with depression and insomnia showed worse immediate word recall in WMT compared to the Insomnia group and both control groups, but they made fewer errors on the TMT.

### 2.3. Brain Demyelination in Post-COVID Patients with Insomnia and Depression

**Figure 1:**
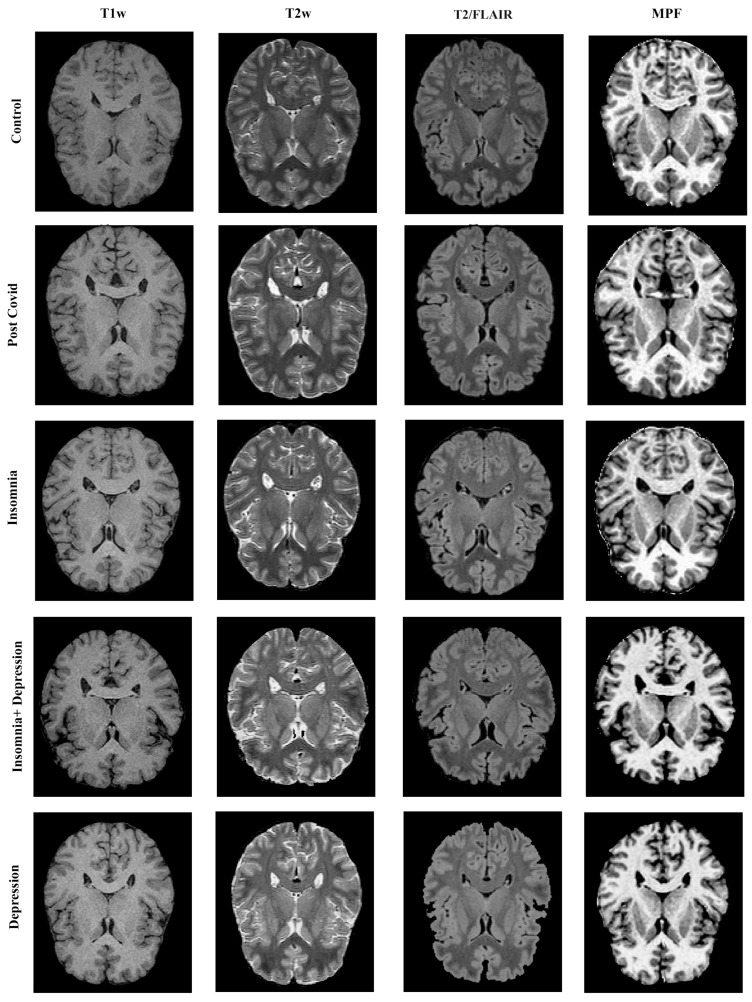
Example T1w, T2w, and T2/FLAIR images and MPF maps of the participants from the Control (40 years, female), PostCovid (42 years, female), Insomnia (43 years, female), InsDep (43 years, female), and Depression (44 years, female) groups. In the figure, window levels were set as following: T1w—0–1000, T2w—0–500, T2/FLAIR—0–350, T2w—0–190.

The MPF maps and T1-weighted, T2-weighted, and T2/FLAIR images of the study participants are shown in Figure 1. No visible differences between participants from different groups were found.

**Figure 2:**
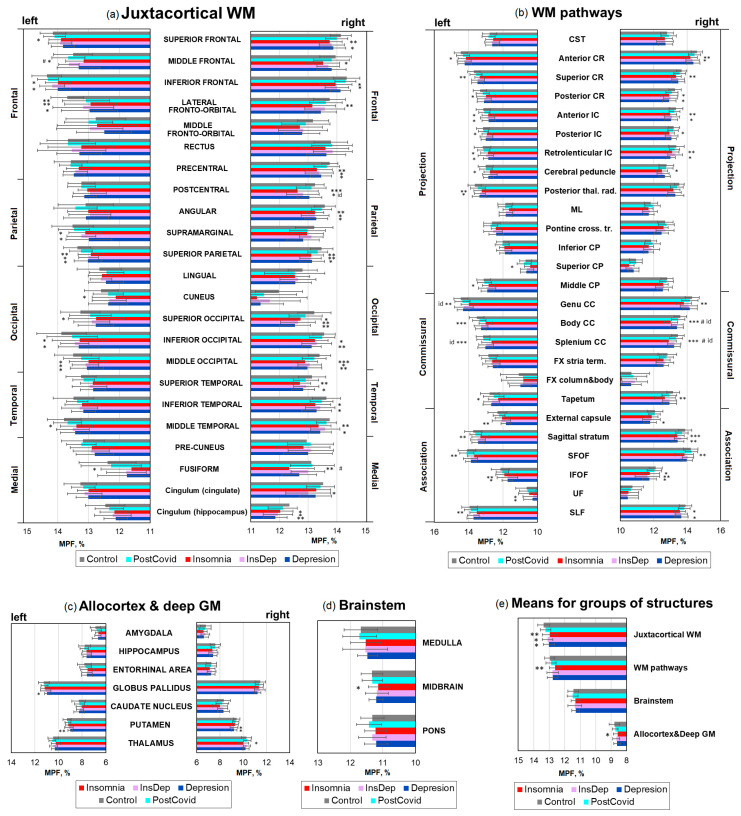
Differences in MPF measurements between Insomnia, InsDep, Depression, PostCovid, and Control groups for separate brain regions: (**a**) juxtacortical WM, (**b**) WM pathways, (**c**) allocortex and deep GM, (**d**) brainstem and (**e**) means for groups of structures. Significant differences relative to controls: *—*p* < 0.05; **—*p* < 0.01; ***—*p* < 0.001. Significant differences relative to the InsDep group: #—*p* < 0.05. Significant differences between the Insomnia and Depression groups: id—*p* < 0.05. Error bars correspond to SD.

**Figure 3:**
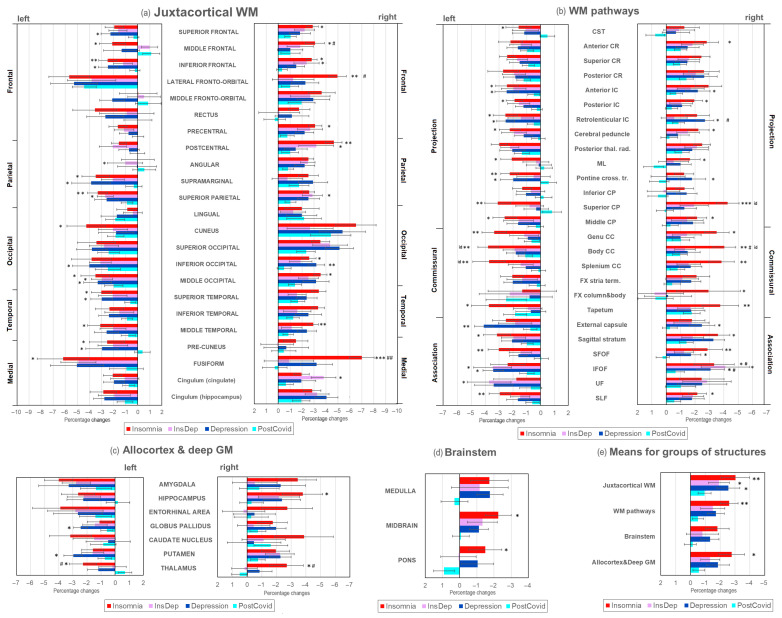
Regional percentage changes in MPF measurements for Insomnia and PostCovid groups relative to the Control group: (**a**) juxtacortical WM, (**b**) WM pathways, (**c**) allocortex and deep GM, (**d**) brainstem, and (**e**) average values for groups of structures. Significant differences relative to the PostCovid group: *—*p* < 0.05; **—*p* < 0.01; ***—*p* < 0.001. Significant differences relative to the InsDep group: #—*p* < 0.05; ##—*p* < 0.01. Significant differences between the Insomnia and Depression groups: id—*p* < 0.05. Error bars correspond to SD.

**Figure 4:**
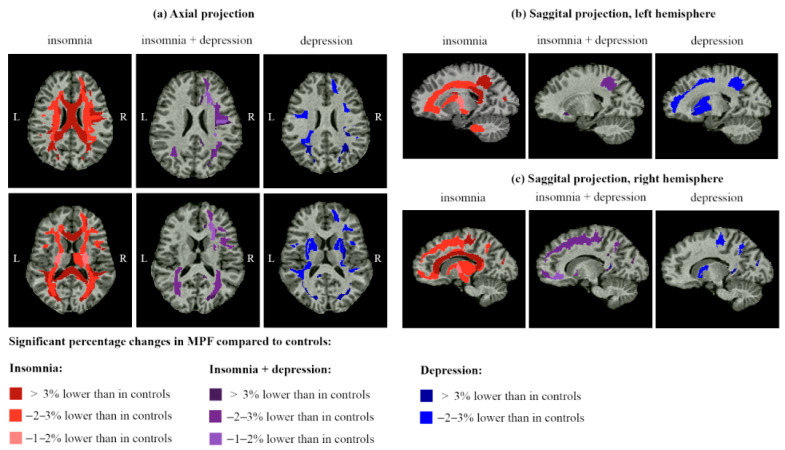
Regions of significant (*p* < 0.05 ÷ 0.001) decrease in MPF for the Insomnia, InsDep, and Depression groups compared to controls: axial views (**a**) and sagittal views of the left (**b**) and right (**c**) hemispheres.

Quantitative MPF measurements showed a significant age- and sex-adjusted decrease in brain myelination compared to the Control group in patients of all experimental groups (Insomnia, InsDep, and Depression) in comparison with healthy controls (Figure 2). However, no significant differences in MPF between the PostCovid and Control groups were found. The age covariate was significant for most brain structures, whereas the sex covariate, as well as the sex × group interaction, were not significant. Figure 3 shows percentage differences in MPF for the Insomnia, InsDep, Depression, and PostCovid groups relative to the controls. Figure 4 summarizes the results presented in Figure 2 and Figure 3 and shows a significant percentage decrease in MPF on brain slices for the experimental groups. In all figures, the color scheme corresponds to the following groups: red—the Insomnia group; blue—the Depression group; purple—the InsDep group; blue-green—the postCovid group; and grey—the Control group.

The most remarkable decrease in MPF compared to controls was observed for the Insomnia group. For this group, a significant decrease in MPF relative controls was observed in 28 out of 46 (61%) explored juxtacortical WM regions (Figure 2a); 28 out of 52 (54%) WM pathways (Figure 2b); 1 out of 14 (7%) deep GM and allocortical regions (Figure 2c); and 1 out of 3 (33%) brainstem regions (midbrain, Figure 2d). In total, 58 out of 115 (50%) studied brain structures demonstrated lower MPF. The average percent decrease (Figure 3e) was significantly higher for the Insomnia group compared to the PostCovid group in juxtacortical WM (−3.03 ± 0.95% vs. 0.95 ± 0.44%), WM pathways (2.61 ± 0.50% vs. 0.50 ± 0.37%), allocortex and deep GM (2.07 ± 0.50% vs. 0.92 ± 0.39%), and brainstem (1.34 ± 0.60% vs. 0.08 ± 0.47%).

The long COVID patients with depression but without insomnia (Depression group) showed a significant MPF decrease compared to controls in fewer structures (Figure 2, 31 of 115, 27%) than for the Insomnia group: 17 out of 46 (37%) juxtacortical WM regions, 11 out of 52 (21%) WM pathways, and 3 out of 14 (21%) deep GM and allocortical regions. In the Depression group, the percentage decrease in MPF compared to the Control and PostCovid groups was generally smaller than in the Insomnia group, except for a few structures: the external capsule, IFOF, UF, supramarginal, cingulum, and inferior and superior occipital WM. In several structures, the percentage MPF decrease in the Depression group was significantly smaller than in the Insomnia group: the superior CP, body, and splenium of CC.

The InsDep group showed smaller but significant MPF decrease compared to the Insomnia and Depression groups (16 out of 115, 14%): 11 out of 46 (24%) juxtacortical WM regions (Figure 2a), 4 out of 52 (8%) WM pathways (Figure 2b), and 1 out of 14 (7%) deep GM and allocortical regions. The magnitude of the MPF decrease was also less than for the other two groups, except for the UF, right cingulum WM, and IFOF, in which this parameter is significantly higher compared to the other groups (−4.11 ± 1.59% vs. 13.32 ± 1.40% and −3.08 ± 0.97%).

The detailed description of the quantitative differences in MPF in different brain structures is summarized in the Supplementary Materials.

### 2.4. Associations Between Neuropsychiatric Parameters and Brain Myelination in Post-COVID Patients with Insomnia and Depression

**Figure 5:**
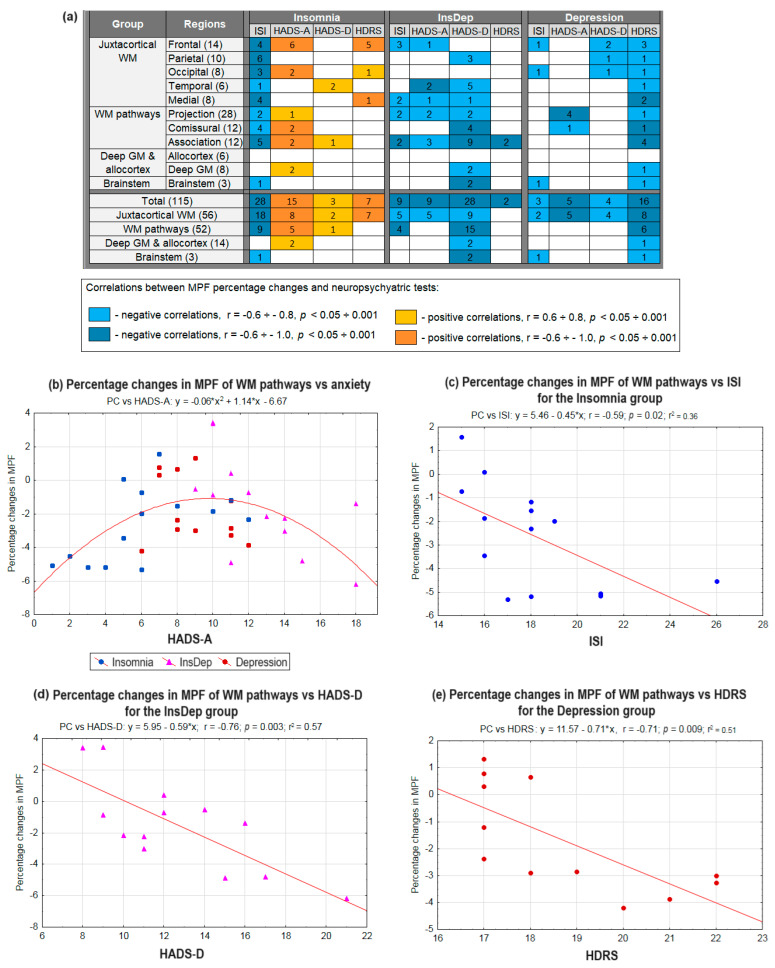
Age-controlled partial correlations between ISI, HADS, HDRS indexes, and percentage changes (PCs) in MPF relative controls for the Insomnia, InsDep, and Depression groups: (**a**) the number of significant correlations in different brain regions, (**b**) quadratic model of anxiety HADS index (HADS-A) with PC in WM pathways for the patients with insomnia and depression, (**c**–**e**) linear regression between MPF percentage changes in WM pathways and (**c**) ISI in the Insomnia group, (**d**) HADS depression index (HADS-D) in the InsDep group, and (**e**) HDRS scores in the Depression group.

The results of the age-controlled regression analysis are shown in Figure 5 and Supplementary Table S1. Significant negative partial correlations of the ISI score with the percentage change in MPF (MPF PC) for separate brain regions were found for all groups. However, the number of such correlations was substantially higher for the Insomnia group than for the InsDep and Depression groups (Figure 5a, Supplementary Table S1). Decreased MPF PC correlated with a higher insomnia index in 28 structures, including the juxtacortical WM regions, tracts, and brainstem. Decreased MPF PC was correlated with a higher insomnia index in 28 structures, including the juxtacortical WM regions, tracts, and brainstem. Moreover, for the Insomnia group, ISI was significantly correlated with the mean MPF PC in the juxtacortical WM (r = −0.62, *p* < 0.01) and WM pathways (r = −0.59, *p* < 0.05, Figure 5c).

Significant negative linear correlations between MPF PC and depression-related scores (HADS-D, HDRS) were found in the InsDep and Depression groups, while correlations of those parameters were positive in the Insomnia group (Figure 5a; Supplementary Table S1). For the InsDep and Depression groups, depression-related parameters showed negative relationships with mean MPF PC (Figure 5d,e) in the juxtacortical WM (r = −0.61, *p* < 0.05, Depression group) and WM pathways (*p* < 0.01, r = −0.71 for the InsDep group and r = −0.76 for the Depression group), while a positive correlation between anxiety and mean MPF PC in the juxtacortical WM (r = 0.56, *p* < 0.05) was found.

Since significant correlations of opposite signs between MPF PC and anxiety/depression indices were found for the Insomnia group versus the other groups, this association was investigated within a quadratic model. The best model fit was obtained for the relationship between the HADS anxiety score and MPF PC in the WM pathways (R = 0.52, *p* < 0.001) (Figure 5b) and juxtacortical WM (R = 0.57, *p* < 0.001). For both juxtacortical WM and WM tracts, quadratic models showed significance for all three coefficients of the equation (*p* < 0.01÷0.001) and the overall model significance (*p* < 0.01 for juxtacortical WM and *p* < 0.01 for WM tracts). The peak values (parabola maximum) of the HADS_A score within the resulting model were 9.6 with MPF = −1.3% for the WM pathways and 10.5 with MPF = −1.3% for the juxtacortical WM. Similar quadratic models of the relationships between MPF PC in the juxtacortical WM and depression-related parameters approached significance (*p* < 0.1). The quadratic model for the HADS-D showed all three significant equation coefficients (*p* < 0.05), and the model for the HDRS showed two significant coefficients (*p* < 0.05), but neither fit was significant overall according to the F-test.

### 2.5. Associations of the Levels of BDNF, Anti-S100, Anti-SARS-CoV-2, and Myelin-Specific Autoantibodies in Blood Plasma with Neuropsychiatric Parameters and Demyelination

The levels of anti-myelin (anti-MBP and anti-PLB), neuroactive (BDNF and anti-S100), and anti-SARS-CoV-2 (S-IgG) antibodies in the blood serum are presented in Table 4. Significant differences were found only for the anti-PLP antibody: Its level was higher in the Insomnia group and lower in the PC group in comparison to the controls. In addition, the anti-PLP level was significantly higher in the Insomnia group compared to the Depression group. The levels of anti-spike IgG in the patients of the InsDep and Depression group were higher than those of the controls, but these differences were only near significant (*p* < 0.1). In the Control group, the level of anti-spike IgG was non-zero, and relatively high levels of antibodies were found in some participants who were not vaccinated and did not have COVID-19. Some patients who had severe COVID-19 or three episodes of COVID-19 before this study had zero anti-spike IgG levels. No differences between groups in anti-MBP, BDNF, and anti-S100 levels were found.

Significant correlations of neuropsychiatric scales and demyelination were found with anti-PLP, anti-MBP, BDNF, and S-IgG antibodies (Table 5). In the total sample of patients, a weak but significant positive correlation of anti-PLP autoantibodies with symptoms of insomnia and depression was observed. A significant negative correlation between S-IgG and ISI for the Insomnia group was found. The BDNF levels showed positive correlations with the severity of insomnia and depression in the InsDep and Depression groups and negative correlations in the Insomnia and PostCovid groups.

Significant negative correlations between the anti-PLP and anti-MBP autoantibody levels and demyelination were found for the total sample of patients and all groups separately, except for the Depression group, for which these correlations were near significant (*p* < 0.01). The S-IgG levels showed significant positive correlations with demyelination for the total sample of patients and for the Depression and PostCovid groups. BDNF levels showed significant negative correlations with demyelination for the total sample of patients and the PostCovid group.

## 3. Discussion

### 3.1. Summary of Results

To our knowledge, this is the first study evaluating an association between insomnia–depression comorbidity and brain demyelination as a consequence of COVID-19. Surprisingly, the greatest demyelination was observed in patients with insomnia but without depression. The least demyelination was found in patients with combined insomnia and depression. The medium demyelination level was detected in the group of patients with depression after COVID-19. The associations between demyelination and neuropsychiatric scales differed across the three experimental groups: Demyelination was more associated with the ISI in patients in the Insomnia group and with depression-related scales in patients in the Depression and InsDep groups. A decline in cognitive performance was found for all experimental groups, but this decline was detected by different tests. Significant associations were found between high levels of serum myelin-specific autoantibodies and demyelination in long COVID patients, as well as the severity of insomnia and depression.

### 3.2. Demyelination and Cognitive Performance in Long COVID Patients with Insomnia

The post-COVID patients of the Insomnia group showed the most prominent demyelination compared to the other groups. For this group, the mean MPF and mean MPF PC were significantly lower in the superficial WM, WM tracts, and allocortical and deep GM than in the Control and PostCovid groups. The following types of WM pathways and anatomically associated brain structures showed the greatest demyelination: (1) commissural pathways—the CC and adjacent tapetum; (2) projection pathways—corticospinal (precentral WM, CR, IC, CST, cerebral peduncles, and midbrain), spinothalamic (postcentral WM, ML thalamus, and midbrain), and cerebellar (superior, middle, and inferior CP, and midbrain); (3) association pathways—the sagittal stratum, SLF and SFOF (often considered as an elongation of the SLF [66]), fusiform, cuneate, and the frontal WM connecting them.

We found an association between myelin loss in the commissural pathways and insomnia, specific to the Insomnia group. This association was demonstrated by other studies in patients with agenesis of the corpus callosum [67,68] and split-brain patients [69]. It was found that agenesis of the corpus callosum affects sleep quality and the shortening of the rapid eye movement (REM) cycle rather than sleep duration [67]. In patients with total callosotomy, greater asymmetry was found for non-rapid eye movement (NREM) epochs, with a relative overall predominance of the right over the left hemisphere. In our study, the greatest MPF decrease in the Insomnia group was also found in the right hemisphere. The reduced connectivity of the CC in patients with insomnia has also been shown in DTI studies [19,21,34,37]. The study by Reyes et al. showed that the long sleeper group (>8.0 h) had lower fractional anisotropy in CC, CR, SLF, and thalamic radiation, which is indicative of a reduction in sleep quality rather than sleep duration and is consistent with our results. It can be hypothesized that demyelination of commissural pathways might be related to/sleep disturbances in the Insomnia group, while extensive demyelination of associative and projection pathways is more associated with cognitive impairment in this group. For the Insomnia group, in contrast to other groups, a decrease in the MoCA test and high-interference (CW) Stroop task was found. Numerous studies (reviewed in [70]) indicate a potentially bidirectional relationship between insomnia and cognitive decline: insomnia may increase the risk of cognitive decline, including through white matter damage, while cognitive decline may contribute to insomnia, further impairing sleep quality.

### 3.3. Demyelination and Cognitive Performance in Long COVID Patients with Depression

The Depression group showed a generally smaller MPF decrease than the Insomnia group, but it was greater than in the InsDep group. In the Depression group, the mean MPF decrease significantly differed from controls only in juxtacortical WM, but some superficial regions exhibited greater MPF decrease compared to the Insomnia group: inferior and superior occipital, supramarginal, and cingulum WM. The mean decrease in MPF relative to controls was not significant for the WM pathways and deep GM, unlike the Insomnia group, but the MPF PC in the external capsule, putamen, left IFOF, UF, and globus pallidus exceeded similar values in the Insomnia group. The external capsule contains the claustrocortical fibers and corticocortical association bundles IFOF and UF, which connect the frontal lobe with the temporal, occipital, and parietal lobes and play a critical role in language semantic processing, goal-oriented behavior, visual switching tasks, and emotion responses [71,72,73,74,75,76,77]. The key role of these associative pathways in MDD has been demonstrated in our recent study [50], as well as many others [78,79,80,81,82,83,84,85,86]. Demyelination of the parahippocampal cingulum was also found in patients of the Depression group in our study. The parahippocampal cingulum is an important part of the limbic system, and it connects the parahippocampal gyrus with the medial temporal lobe; it is linked with learning, episodic memory, executive control, and emotional functions [87]. Changes in the cingulum WM microstructure were found in MDD conditions [88] and other neuropsychiatric and neurocognitive disorders [87]. In our study, only patients in the Depression group performed more slowly on the TMT and the Stroop word recall tasks. This decrease might be related to IFOF and cingulum demyelination due to its involvement in language processing, visuospatial integration, executive control, and goal-orienting behavior.

### 3.4. Demyelination and Cognitive Performance in Long COVID Patients with Comorbid Insomnia–Depression

Unexpectedly, demyelination in the post-COVID patients with comorbid insomnia and depression (InsDep group) was less pronounced in comparison to the post-COVID patients with insomnia or depression alone. Despite less severe demyelination, patients in the InsDep group had the highest scores on both insomnia and depression scales, reported the greatest number of post-COVID symptoms, and showed the worst immediate recall in the WMT. Mean MPF values in the InsDep group were significantly lower than those of the controls only in juxtacortical WM, but the magnitude of this decrease was smaller than in the other two groups. Only for a limited number of structures was the percentage of MPF decrease greater than in the other groups: bilateral IFOF, UF, and right cingulum. These results further support the hypothesis of IFOF, UF, and cingulum demyelination in the development of depression.

The correlation patterns between demyelination and insomnia/depression scales may provide clues to understanding these differences. The negative associations between demyelination and both depression and insomnia were shown in the InsDep group, while in the Insomnia group, MPF PC was negatively correlated with insomnia but positively correlated with anxiety/depression indices. In the total sample, a significant quadratic relationship was found between demyelination and anxiety.

Although we did not directly measure the level of arousal in patients, a positive correlation between anxiety and arousal level can be assumed, because the link between anxiety and neuronal activation is well established [89,90]. In turn, increased axonal firing promotes myelin thickening [91] and remyelination [92] due to glutamate, GABA, BDNF, NRG1 action on the oligodendrocytes and oligodendrocyte precursors, and other molecular mechanisms [92]. However, hyperarousal disrupts this balance and promotes the increased myelination of a very limited number of conducting pathways, while weakening the maintenance and remyelination of others [93]. Regarding the quadratic relationship we found between myelination and anxiety levels, it can be hypothesized that in the group of patients with insomnia, low levels of anxiety and arousal hinder remyelination, while an increase in arousal levels may accelerate this process. In the InsDep group, average anxiety and arousal levels promote remyelination, but hyperarousal decreases it. These assumptions lead to the therapeutic strategy of keeping arousal level optimal: Both hypoarousal and hyperarousal negatively affect myelination. In our study, the quadratic model of the relationship between myelination and anxiety level was obtained using a small sample and requires further research, which could include psychometric tests capable of objectively assessing the level of arousal.

In the InsDep group, demyelination was greatest in IFOF, UF, and the cingulum bundle. These are corticocortical association pathways that integrate information from the frontal, occipital, temporal, and parietal lobes. The cortex has an inhibitory effect on the underlying structures, and an imbalance of excitation–inhibition towards the subcortical structures can cause hyperarousal [94]. We hypothesize that hyperarousal may be a primary cause of insomnia for the Depression and InsDep groups; greater demyelination of the IFOF, UF, and cingulum in the InsDep group might cause more evident hyperarousal and insomnia (negative correlation with anxiety). Recent studies found increased subcortical brain activity in anxious but not depressed individuals [90].

It is likely that the Insomnia group has other mechanisms for the development of insomnia. Extensive demyelination in the CC, projection tracts, and other structures after COVID-19 is associated with disrupted sleep patterns and cognitive decline, which mutually reinforce each other. Low arousal levels in the Insomnia group only hinder active remyelination; therefore, we observed the highest demyelination in these patients.

### 3.5. Specificity of WM Microstructure Changes for Insomnia and Depression After COVID-19

Changes in WM microstructure as a consequence of COVID-19 have already been shown in many DTI studies [55,56,57,58,59,60,61,62]. The systematic review by Nelson et al. [55] showed that the most common findings in long COVID patients were lower WM integrity in the CR, CC, SLF, IFOF, SFOF, posterior thalamic radial area, external capsule, and cingulate gyrus, which is consistent with our study. Several studies reported mixed higher and lower anisotropies with a more widespread lower anisotropy, and only one study reported higher anisotropies in post-COVID patients [55]. Unfortunately, very few of the above studies take into account the specific symptoms in patients with long COVID. This likely explains some of the inconsistency in the results.

It is well known that the widely used DTI method can only assess myelin indirectly due to the dependence on diffusion direction in different regions [46]. In the present study, we used the quantitative MPF mapping method highly specific to myelin content, which is confirmed by histological validation in animal studies [39,40,95,96] and numerous clinical approaches [48,49,50,51,53,97].

Only three published studies (including our [50]) examined WM changes in relation to depression in long COVID patients. Cui et al. [57] investigated DTI parameters and their correlations with psychological scores of patients with severe depression after two COVID-19 infections and patients with mild or moderate depression after one COVID-19 infection as the control group. The patients with severe depression showed decreased FA in the bilateral CST, anterior thalamic radiation, and right SLF. Insomnia- and depression-related psychological scores were negatively correlated with the FA of these WM tracts. The study by Benedetti et al. [64] investigated associations between post-COVID self-rated depression, brain myelination (assessed with DTI), and functional connectivity (assessed with resting-state fMRI). The self-rated severity of psychopathology was negatively associated with AD in several WM tracts: the CR, SLF, ILF, external capsule, and anterior thalamic radiation. Inflammatory markers were also correlated with DTI measures of WM microstructure. Unfortunately, a healthy control group was not included in the above studies, which precluded a meaningful comparison of these studies with our results.

The study of Qin et al. [63] investigated WM changes and inflammation in COVID-19 patients with insomnia. They found that individuals with sleep disorders had specific differences in DTI parameters, such as significantly lower FA and higher RD and AD in the CC, CR, thalamic radiation, CST, forceps, and right ILF and IFOF. These WM abnormalities and IL-1β levels correlated with insomnia scores. Three months after recovery, insomnia-related scores improved slightly, but depression- and anxiety-related scores remained unchanged. However, the significant changes in DTI measures between the acute and post-COVID phases were found for only two structures: the left CST showed an increase in FA, whereas the right IFOF showed a decrease in FA. These findings are important for the interpretation of our results. First, a study by Qin et al. showed the association between inflammation, insomnia, and WM damage in multiple tracts, including the CC, CR, thalamic radiation, CST, and IFOF in the acute phase of COVID-19, which is consistent with our results. Second, FA decrease was revealed in the right IFOF even after recovery and in the absence of improvement in depression and anxiety scores. These results confirmed that the IFOF is a key tract responsible for the persistence of depression in long COVID patients.

The study of Rouen et al. [98] found no significant differences between long COVID patients with insomnia and patients with chronic insomnia but without COVID-19. This means that our findings may be important not only in the context of post-COVID complications but also for understanding the causes of insomnia itself and its connection to depression.

### 3.6. Autoimmunity as a Possible Mechanism of Demyelination in Long COVID

Significant differences in antibody levels between groups were found only for anti-PLP: The highest level was observed in patients with insomnia, who also showed the highest demyelination. Additionally, high levels of anti-PLP antibodies were associated with both the persistence of insomnia/depression symptoms and demyelination. The levels of anti-MBP antibodies showed a similar trend. The role of autoimmunity in acute COVID-19 and long COVID was demonstrated in numerous studies [99,100,101,102,103]. Almulla et al. [103] showed that myelin-specific anti-MBP and anti-MOG IgG are among the best predictors of long COVID and, in particular, affective symptoms in long COVID. Chang et al. [99] revealed that SARS-CoV-2 causes the development of new-onset IgG autoantibodies in 50% of hospitalized COVID-19 patients. This subset of autoantibodies targeting traditional autoantigens or cytokines positively correlated with immune responses to SARS-CoV-2 proteins. However, we found correlations of opposite signs for myelin-specific autoantibodies and anti-spike IgG. Moreover, our results regarding anti-spike IgG levels are consistent with previous studies that showed the protective role of anti-SARS-CoV-2 antibodies in the development of long COVID symptoms [104,105]. In our study, high anti-spike IgG levels positively correlated with MPF PC and negatively correlated with the severity of depression and insomnia. This discrepancy may be due to the greater role of the nucleocapsid protein of SARS-CoV-2 rather than the anti-spike protein of SARS-CoV-2 in the association between anti-PLP and demyelination. Lake et al. [102] found a significant overlap between the nucleocapsid protein of SARS-CoV-2 and PLP, and this overlap may have critical implications for T cell responses both in long COVID and multiple sclerosis. Overall, our results confirmed the important role of serum anti-PLP and anti-MBP antibodies in post-COVID demyelination.

We found no differences between groups in BDNF and anti-S100 levels, but significant correlations between BDNF levels and the severity of insomnia and depression/anxiety were observed. These correlations were negative in the Insomnia and PostCovid groups and positive in the Depression and InsDep groups. Additionally, BDNF levels negatively correlated with demyelination in the total sample of patients and the PostCovid group. This complex pattern of cross-relationships between BDNF levels, neuropsychiatric scores, and demyelination likely reflects the dual influence of BDNF on post-COVID complications: through its direct effects on neurons and through neuroinflammation. It was shown that elevated BDNF levels are associated with microglia activation and pro-inflammatory cytokines release, but prolonged inflammation leads to a decrease in neurotrophin levels [106]. In contrast, a decrease in BDNF levels was found in the acute phase of COVID-19 [107,108], while an increase in BDNF levels was observed in patients with long COVID [109,110]. Both insomnia [111] and depression [112] are usually associated with lower serum BDNF levels in most studies, but their relationship with neuroinflammation in these patients remains poorly understood. Future research will clarify the complex associations between BDNF, neuropsychiatric symptoms, and demyelination.

### 3.7. Study Limitations

The main limitation of the current study is its small sample size. There was also a slight imbalance in the age and gender of the enrolled participants: The InsDep group differed significantly from other groups in gender enrollment. The average age in the Insomnia group was slightly higher than in the other groups, although the difference was not statistically significant. To adjust for the influence of these factors, we used gender and age as covariates in the statistical analysis. Larger and more gender- and age-balanced samples of long COVID patients could yield more reliable results. We enrolled relatively young participants from 18 to 60 years old; older individuals with long COVID were not studied. All patients were diagnosed with clinical depression and/or insomnia for the first time after COVID-19. However, we cannot fully guarantee that the occurrence of these conditions was not influenced by sociodemographic factors associated with the pandemic. We used a limited number of neuropsychiatric scales (HDRS, HADS, and ISI) to assess the severity of insomnia and/or depression. Since patients were not examined by neurologists and psychiatrists before COVID-19, we cannot guarantee the absence of subclinical manifestations of insomnia or depression in patients before this infection.

The present study is exploratory. The patterns we have observed require further clarification using larger samples of patients with insomnia and depression.

## 4. Materials and Methods

### 4.1. Study Participants

The participants for this study, including patients with long COVID (n = 76) and healthy controls (n = 22), were recruited from September 2022 to June 2023. Patients with post-COVID complications were recruited by neurologists at the Medica Diagnostic and Treatment Center and psychiatrists at the Mental Health Research Institute (Tomsk, Russia). To be eligible, participants were required to meet the following criteria: (1) previously tested positive in the COVID-19 PCR test and have persistent post-COVID complications (except for the Control group); (2) aged from 18 to 60 years. Participants were excluded from this study if they had neurological or psychiatric diagnoses prior to COVID-19, acute infection or somatic diseases, pregnancy, a history of traumatic brain injury, contraindications or intolerance to the MRI procedure, or self-withdrawal. The Control group included healthy volunteers without prior COVID-19 history. An informed consent form was signed by all participants who met the inclusion and exclusion criteria. This study was performed in accordance with the guidelines of the Declaration of Helsinki. The Ethical Committee of the Mental Health Research Institute (protocol No 15 dated 25 August 2022) and the Bioethics Committee of Tomsk State University (No 12 dated 6 June 2022) approved this study.

All recruited participants were initially diagnosed with long COVID symptoms, including insomnia and depression. All diagnoses were carried out prior to study enrollment.

The study participants were assessed by a clinical psychologist and a psychiatrist using several standard psychometric tests, including the Insomnia Severity Index (ISI) [113], the Hospital Anxiety and Depression Scale (HADS) [114], and the Hamilton Depression Rating Scale (HDRS) [115].

The ISI test (total score ≥ 15) was performed by a clinical psychologist and was confirmed by a neurologist based on a clinical interview. This test is used to determine the nature, severity, and impact of insomnia on daily life over the past two weeks. Specifically, the questionnaire rates each of the following parameters from 0 to 4: difficulty falling asleep, difficulty of sleep maintenance, early awakening problems, satisfaction with current sleep pattern, interference with daytime functioning, noticeability of sleep difficulties by others, and distress caused by sleep abnormalities. The total score was considered as follows: no insomnia (0–7); mild insomnia (8–14); moderate insomnia (15–21); and severe insomnia (22–28) [113].

Depression was diagnosed by a psychiatrist according to clinical criteria for major depressive disorder (MDD), as described elsewhere [50]. This study only included patients whose symptoms of depression appeared after coronavirus infection and were absent before it. The Hospital Anxiety and Depression Scale (HADS) [114] was used for the screening assessment of anxiety and depression using the HADS-A and HADS-D subscales correspondingly. Each subscale ranges from 0 to 21. All participants were assessed by a clinical psychologist, and those who had scores of 8 or higher were assessed by a psychiatrist. The diagnosis of clinical depression was made by a psychiatrist based on a structured clinical interview according to ICD-10 and clinical and psychometric examinations. The severity of the current depressive episode was assessed using the Hamilton Rating Scale for Depression (HDRS) [115] before the start of drug therapy. The severity of depression was assessed according to the HDRS total score: no depression (0–7); mild depression (8–16); moderate depression (17–23); and severe depression (≥24).

The groups were formed as follows: (1) InsDep group (n = 13)—patients with diagnosed both insomnia and depression; (2) Insomnia group (n = 14)—patients with insomnia but without depression; (3) Depression group (n = 12)—patients with depression but without insomnia; (4) PostCovid group (n = 32)—patients with post-COVID complications without insomnia and depression; (5) Control group (n = 22)—patients who have not had COVID-19. The demographic characteristics of participants are presented in Table 6. The groups did not differ significantly in age, education, or severity of COVID-19. The InsDep group differed significantly from the other groups in the percentage of men and women; therefore, the covariate “gender” was included in the statistical design.

All recruited participants answered the COVID-19 questionnaire [50] and underwent MRI scanning and neuropsychological testing 1–2 days after MRI. Five participants with newly diagnosed brain pathologies (cavernoma and vascular anomalies) were excluded from further analysis.

### 4.2. Neurocognitive Testing

In addition to the tests required for group assignment (ISI, HADS, and HDRS), as described above, study participants were assessed using several standard psychometric cognitive tests: the Montreal Cognitive Assessment (MoCA) [116], Word Memory Test (WMT) [117,118], Trail Making Test (TMT) [119], and Stroop Color Word Test (SCWT) [120,121].

Participants were presented with the Russian variant [122] of the Montreal Cognitive Assessment (MoCA) test [116], version 7.1 [123], for global assessment of cognitive function. A total score of 25 or less was defined as cognitive impairment, while 25 or more was considered normal [116,122].

The Russian version of the classical Stroop task [120,121], modified by Cousijn et al. [124], was used for cognitive control assessment. This test consisted of three tasks: the W, C, and CW conditions. The word condition (W) included the presentation of the words meaning four colors (“cиний” (blue), “зeлeный” (green), “кpacный” (red), “жeлтый” (yellow)) printed in black font. Participants were asked to read the words as quickly as possible. The color condition (C) included solid-color hexes (blue, green, red, or yellow); participants were asked to name the color. The word-color (WC) condition involved the presentation of words denoting four colors printed in a mismatched color (e.g., the word “blue” printed in red ink). The participants were asked to name the font color. The total time spent completing each of the three conditions was measured.

The Russian version [125] of the Word Memory Test (WMT) [117,118] included the presentation of printed 10 Russian unrelated words. The participant was asked to read and remember each word. The immediate recall of the words shown was assessed. The psychologist then attempted to help the participant reproduce the missing words using associations (assistance). After approximately 15 min, the participant was asked to recall as many previously memorized words as possible. After this, the psychologist tried to recall missing words using associations. The assigned scores corresponded to the number of correctly reproduced words, both immediately (0–10 scores) and in the delayed period (0–10 scores), with and without the help of a psychologist.

The Trail Making Test (TMT), part A [119], involved connecting 25 numbered circles in ascending order. The test was scored based on the time taken to complete the task and the number of errors.

### 4.3. MRI Data Acquisition

Magnetic resonance imaging was performed using a 1.5 T clinical scanner Magnetom Essenza (Siemens, Erlangen, Germany). To obtain MPF maps [49], the following 3D spoiled gradient-echo pulse sequences were included in the protocol:

- Magnetization Transfer (MT)-Weighted: TR = 20 ms; echo time (TE) = 4.76 ms; flip angle (FA) = 8°; scan time: 5 min 40 s;
- T1-Weighted: TR = 16 ms; TE = 4.76 ms; FA = 18°; scan time: 4 min 32 s;
- Proton Density (PD)-Weighted: TR = 16 ms; TE = 4.76 ms; FA = 3°; scan time: 4 min 32 s.

The additional imaging sequences included the following:

- 3D Fluid Attenuated Inversion Recovery (T2/FLAIR): TR = 5000 ms; TE = 390 ms; TI = 1800 ms;
- 3D T1-Weighted: TR = 16 ms; TE = 4.76 ms;
- 3D T2-Weighted: TR = 3000 ms; TE = 335 ms.

All scans were acquired in the sagittal imaging plane with a field of view of 240 × 240 × 200 mm^3^, voxel size of 1.25 × 1.25 × 1.25 mm^3^, matrix of 192 × 192 × 160, and single signal averaging. The total scanning time was about 35 min.

### 4.4. Image Processing

Three-dimensional MPF maps were obtained using a single-point algorithm with a synthetic reference image [44,126]. Reconstruction of MPF maps was carried out using the previously developed software available at https://www.macromolecularmri.org/ (accessed on 13 December 2025).

**Figure 6:**
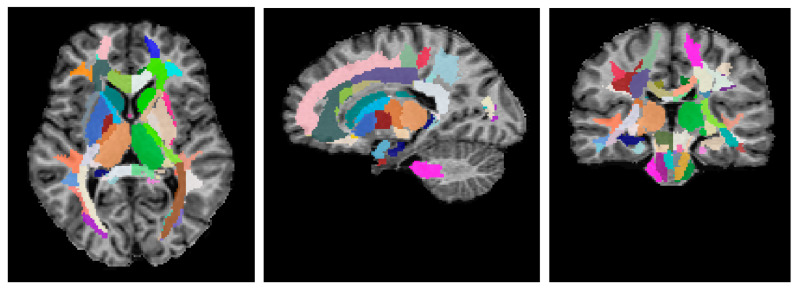
An example of the segmentation of an individual MPF map of the healthy control participant using its registration to the T1 Eve template [129]. Slices in similar axial, sagittal, and coronal projections are shown. Different colors indicate 118 regions of interest (ROIs) for different structures of the juxtacortical WM, WM tracts, brainstem, allocortex, and deep GM.

Advanced Normalization Tools (ANTs) [127,128] and the Eve anatomical atlas [129] were used for regional WM and GM segmentation, as described in [50,51,53]. To obtain individual segmentation, the T1 template images of the Eve atlas were registered to individual MPF maps, and the obtained deformation field was applied to the Type-III Eve atlas segmentation [129]. An example of regional segmentation is presented in Figure 6.

Average MPF values in the right and left hemispheres were calculated using ITK-snap software (version 3.6.0) for the following GM and WM brain structures:

Juxtacortical (superficial) WM: (1) frontal lobe—superior, middle, and inferior frontal; lateral and middle fronto-orbital; rectus; and precentral WM. (2) Parietal lobe—postcentral; superior parietal, angular; supramarginal, cuneus; and lingual WM. (3) Occipital lobe—superior, inferior, and middle occipital WM. (4) Temporal lobe—superior, inferior, and middle temporal WM. (5) Medial surface—pre-cuneus; fusiform; cingulum (parts of cingulate gyrus and hippocampus) WM:

1. WM Pathways and Fasciculi: (1) projection tracts—corticospinal tract (CST); anterior, superior, and posterior corona radiata (CR); anterior, posterior limb, and retrolenticular part of internal capsule (IC); cerebral peduncles; posterior thalamic radiation; medial lemniscus (ML); pontine crossing tract; inferior, superior, and middle cerebellar peduncles (CP). (2) Commissural tracts—genu, body, and splenium of corpus callosum (CC); fornix (FX) (stria terminalis, column, and body); tapetum. (3) Association tracts—superior longitudinal (SL) fasciculus; superior (SFOF) and inferior fronto-occipital (IFOF) fasciculi; uncinate fasciculus (UF); sagittal stratum; external capsule.
2. Subcortical and Allocortical GM Structures: (1) allocortex—amygdala; hippocampus; entorhinal area. (2) Deep GM—caudate nucleus; putamen; globus pallidus; thalamus.
3. Brainstem Structures: medulla; midbrain; pons.

All measurements were obtained for both the left and right hemispheres, except for the brainstem. For these structures, measurements in the left and right hemispheres were averaged. As a result, 115 measurements were obtained in total.

### 4.5. ELISA

Biological samples were collected from the cubital vein of each participant after a 12 h overnight fast using vacuum tubes containing a clot activator. The sedimentation of the clot and cellular components was achieved through centrifugation for twenty minutes at 2000× *g* at 4 °C. Aliquots of the resulting purified serum were stored at −80 °C until subsequent testing. Quantitative assays for IgG antibodies against the myelin basic protein (anti-MBP), proteolipid protein (anti-PLP), and S100 (anti-S100), as well as serum levels of brain-derived neurotrophic factor (BDNF), were conducted utilizing specific ELISA kits from Cloud-Clone Corp. (CCC, Houston, TX, USA) in accordance with the manufacturer’s protocols. The level of Human Anti-SARS-CoV-2 Spike Protein (Trimer) IgG antibodies (S-IgG) in serum was determined using the ELISA Kit from AntibodySystem (BioLab, Wuhan, China). Absorbance at 450 nm for each well was measured with a Varioskan LUX spectrophotometer (Thermo Scientific, Waltham, MA, USA). The calculation of the concentrations of the determined proteins was performed in accordance with the manufacturer’s guidelines.

### 4.6. Statistical Analysis

Statistical analysis was performed using Statistica 10.0 software. Differences between groups in neuropsychological parameters, MPF, and percentage changes (PCs) in MPF for each brain structure were analyzed using one-way analysis of covariance (ANCOVA), with age and gender as covariates, followed by post hoc Fisher LSD tests. The MPF PC relative to the Control group was calculated using the following formula: (MPF_ind_ − MPF_control_)/MPF_control_ × 100, where MPF_ind_ is the individual MPF value for each brain structure, MPF_control_ is the average MPF for the same structure in the Control group. False discovery rate (FDR) correction was performed to prevent false positive results in multiple comparisons. Differences between groups in the gender of participants and severity of COVID-19 were assessed using the chi-square criteria.

Regression analysis was performed for each of the brain structures separately and for the mean MPF measurements for groups of structures (juxtacortical WM, WM pathways, brainstem, allocortex, and deep GM). Age-controlled partial correlations between neuropsychiatric parameters (ISI, HADS-A, HADS-D, and HDRS scores) and MPF PC were calculated for the Insomnia, InsDep, and Depression groups separately. The relationships were examined by fitting linear and quadratic functions. If the fitting of the quadratic function was significant according to T-tests for coefficients and F-criteria for the overall model, the peak value of the neuropsychiatric parameter was calculated from the quadratic equation. Since the distribution of IgG levels differed from normal, the nonparametric Newman–Keuls test was used for post hoc analysis of differences between groups, and the nonparametric Spearman coefficient was used to study correlations.

All tests were considered statistically significant with *p* values less than 0.05.

## 5. Conclusions

Our study is the first to investigate WM damage and change in cognitive performance specific to insomnia, depression, and comorbid insomnia–depression as COVID-19 consequences. Significant impairments in cognitive tests and myelination were found for all studied groups compared to healthy controls and long COVID patients without insomnia and depression. The Insomnia group showed the greatest demyelination among all groups, both in localization and amplitude, which negatively correlated with insomnia severity. Specific characteristics of this group were the demyelination of commissural (CC and tapetum), projection (CR, IC, CST, cerebral peduncles, CP, and ML), some associative pathways (sagittal stratum, SLF, and SFOF), and a number of other structures closely associated anatomically and functionally (thalamus, midbrain, postcentral, precentral, fusiform, cuneate, and frontal WM). The Insomnia group also showed a decrease in the MoCA test and high-interference (CW) Stroop task performance. The levels of serum anti-PLP antibodies were the highest in the Insomnia group and correlated with both the persistence of insomnia/depression symptoms and demyelination.

The Depression and InsDep groups showed a smaller MPF decrease than the Insomnia group; this decrease negatively correlated with depression severity. A characteristic feature of the Depression and InsDep groups was the greater demyelination of the association pathways, IFOF, and UF, compared to the Insomnia group. The Depression group demonstrated a higher overall demyelination level than the InsDep group and the highest MPF decrease in the globus pallidus, putamen, and external capsule. The InsDep group demonstrated the highest level of demyelination in the IFOF, UF, and right cingulum. The patients in the Depression group performed the TMT and the Stroop word recall task more slowly, while the patients of the InsDep group showed worse immediate word recall in the WMT test. We also found a quadratic relationship between demyelination and anxiety levels in patients with insomnia and/or depression. Based on the results, we assume that demyelination in long COVID is associated with high levels of serum myelin-specific autoantibodies, but symptoms of insomnia and/or depression are associated with demyelination of a different set of brain structures. In the absence of depression, insomnia is closely associated with the extensive demyelination of commissural, projection, and some association pathways, which might be related to cognitive decline. In the presence of depression, insomnia due to hyperarousal might be related to demyelination of the corticocortical association pathways, IFOF, and UF and, as a result, an imbalance of cortico-subcortical excitation and inhibition. Future studies of patients with MDD and insomnia, regardless of COVID-19, are needed to confirm or refute these hypotheses.

## Figures and Tables

**Table 1:** Parameters associated with the COVID-19 disease and its consequences.

Parameter	Insomnia	Ins-Dep	Depression	PostCovid	Control
Severity, mild/moderate/severe/critical (%)	57/29/14/0	84/8/8/0	92/8/0/0	77/7/11/5	-
Number of COVID-19 episodes, mean ± SD	1.64 ± 0.63	1.54 ± 0.66	1.75 ± 0.873	1.63 ± 0.79	-
Time after the first COVID-19, months ± SD	21.5 ± 8.9	22.2 ± 7.2	18.3 ± 9.1	22.1 ± 10.5	-
Time after the last COVID-19, months ± SD	14.9 ± 8.5	15.7 ± 10.5	10.3 ± 9.8	15.1 ± 11.5	-
Number of acute symptoms	6.8 ± 1.5	7.4 ± 1.9	7.1 ± 1.8	6.1 ± 2.4	-
Number of post-COVID symptoms	6.9 ± 2.7	8.2 ± 2.1 *	7.8 ± 2.4	6.0 ± 3.0	-
Vaccinated at the time of study (%)	42.9	53.9	100	50.0	68.2

Data are presented as mean ± SD. Significant differences relative to the PostCovid group: *—*p* < 0.05.

**Table 2:** Results of tests related to insomnia and depression.

Test	Parameter	Insomnia	InsDep	Depression	PostCovid	Control
**ISI**	Total score	17.1 ± 2.9 D, PC, C	20.1 ± 4.9 D, PC, C	8.6 ± 2.5 I, ID, PC	5.7 ± 3.6 I, ID, D	6.1 ± 4.9 I, ID
**HADS**	Total score	12.6 ± 7.0 ID, C	25.1 ± 6.3 A	16.7 ± 4.9 I, PC, C	10.4 ± 4.9 I, ID	7.4 ± 3.8 A
	Anxiety	6.9 ± 3.7 D, ID, C	12.7 ± 3.0 A	8.8 ± 2.2 ID, PC, C	6.0 ± 3.7 D, ID, C	4.05 ± 2.44 A
	Depression	6.3 ± 3.9 ID, C	12.7 ± 3.7 A	7.8 ± 4.6 I, PC, C	4.6 ± 2.9 D, ID	3.4 ± 2.4 I, ID, D
**HDRS**	Total score	11.4 ± 4.7 A	21.3 ± 4.0 I, PC, C	18.3 ± 2.4 I, PC, C	8.4 ± 4.4 A	3.5 ± 3.4 A

Data are presented as mean ± SD. Significant differences (*p* < 0.05–*p* < 0.001) relative to the following: A—all groups; C—the Control group; I—the Insomnia group; ID—the Ins-Dep group; D—the Depression group; PC—the PostCovid group.

**Table 3:** The results of cognitive testing.

Test	Parameter	Insomnia	InsDep	Depression	PostCovid	Control
**MoCA**	Total score	25.9 ± 2.8 C	26.6 ± 1.8	26.3 ± 2.5	26.8 ± 1.9	27.6 ± 1.5
**WMT**	Total score	18.6 ± 2.2	17.7 ± 3.1	19.0 ± 1.0	18.9 ± 1.5	19.1 ± 1.3
	Immediate recall	7.5 ± 1.0	6.6 ± 1.3 PC, C	7.6 ± 1.7	7.7 ± 1.3 ID	8.4 ± 1.4 ID
	Immediate recall with assistance	9.7 ± 0.5 ID	8.8 ± 1.5 I, PC, C	9.3 ± 1.3	9.7 ± 0.5 ID	9.8 ± 0.5 ID, D
	Delayed recall	6.3 ± 2.1	6.5 ± 2.4	7.3 ± 1.6	7.0 ± 1.8	7.2 ± 2.1
	Delayed recall with assistance	9.3 ± 0.9	8.8 ± 2.0	9.3 ± 1.2	9.3 ± 1.1	9.2 ± 1.2
**SCWT**	W, time (s)	51.9 ± 6.5	53.3 ± 10.9	57.7 ± 13.6 PC, C	51.8 ± 7.4	51.0 ± 9.1
	C, time (s)	74.0 ± 17.5	65.1 ± 24.2	72.8 ± 19.0	66.9 ± 10.1	65.5 ± 18.7
	CW, time (s)	132.1 ± 27.6 C	117.3 ± 40.4	121.1 ± 30.9	118.8 ± 22.0	114.0 ± 42.7
**TMT**	Processing time, s	37.0 ± 11.1 D	37.5 ± 14.9	46.0 ± 21.1 I, PC, C	32.5 ± 7.9 D	34.1 ± 7.9 D
	Errors, mean ± SD	0.6 ± 0.6 ID	0.1 ± 0.3 I	0.2 ± 0.4	0.4 ± 0.6	0.5 ± 0.7

Data are presented as mean ± SD. Significant differences (*p* < 0.05–*p* < 0.001) relative to the following: C—the Control group; I—the Insomnia group; ID—the Ins-Dep group; D—the Depression group; PC—the PostCovid group.

**Table 4:** The levels of anti-myelin, neuroactive, and anti-SARS-CoV-2 antibodies in serum.

Antibodies	Control	PostCovid	Depression	InsDep	Insomnia
BDNF ng/m	69.73 ± 11.20	64.01 ± 11.64	66.44 ± 11.01	68.54 ± 10.60	71.50 ± 8.48
Anti-S100, ng/mL	7.12 ± 5.15	6.99 ± 3.80	5.93 ± 6.74	8.44 ± 7.37	9.33 ± 8.07
Anti-MBP ab ng/mL	2.41 ± 3.04	10.28 ± 18.24	17.46 ± 40.38	0.97 ± 1.61	25.73 ± 45.10
Anti-PLP, ng/mL	1.33 ± 0.22	0.99 ± 0.54 **	1.04 ± 0.85 #	1.44 ± 0.69	1.77 ± 1.16 *
IgG, ng/mL	1.63 ± 3.84	2.60 ± 3.81	4.78 ± 5.58	4.49 ± 5.65	1.76 ± 3.94

Data are presented as mean ± SD. Significant differences relative to the Control group: *—*p* < 0.05, **—*p* < 0.01. Significant differences between the Insomnia and Depression groups: #—*p* < 0.05.

**Table 5:** The associations of anti-myelin, neuroactive, and anti-SARS-CoV-2 antibodies with neuropsychiatric testing and brain demyelination.

Parameters		All Patients	Insomnia	InsDep	Depression	PostCovid
Neuropsychiatric scales	ISI	Anti-PLP (0.30 *)	S-IgG (−0.63 *)	BDNF (0.59 *)	BDNF (0.61 *)	
	HADS-A				BDNF (0.71 *)	BDNF (−0.43 *)
	HADS-D		BDNF (−0.74 *)			
	HDRS	Anti-PLP (0.31 *)		BDNF (0.58 *) S-IgG (−0.68 *)		BDNF (−0.53 *)
MFP PC	Juxtacortical WM	Anti-PLP (−0.34 *)			S-IgG (0.85 **)	Anti-PLP (−0.42 *)
	WM pathways	Anti-PLP (−0.30 *)			S-IgG (0.86 ***)	Anti-PLP (−0.46 *)
	Allocortex and deep GM	Anti-PLP (−0.30 *) BDNF (−0.29 *) S-IgG (0.26 *)	Anti-MBP (−0.63 *)			Anti-PLP (−0.58 **)
	Brainstem	S-IgG (0.27 *)	Anti-MBP (−0.64 *)	Anti-MBP (−0.68 *)		BDNF (−0.44 *)

Only significant coefficients of correlation are presented for specific antibodies. Significant Pearson’s coefficients of correlation for anti-PLP, anti-MBP, and BDNF: *—*p* < 0.05; **—*p* < 0.01. Significant Spearman coefficients of correlation for IgG: *—*p* < 0.05; **—*p* < 0.01; ***—*p* < 0.001.

**Table 6:** The demographic characteristics of participants in this study.

Parameter	Insomnia	InsDep	Depression	PostCovid	Control
Sample size	14	13	12	32	22
Male (%)/Female (%)	3(21)/11(79)	2(15)/11(85) *	2(17)/10(83)	17(39)/27(61)	11(50)/11(50)
Age, years ± SD	45.2 ± 8.9	38.5 ± 12.6	35.3 ± 15.2	41.6 ± 10.9	40.6 ± 11.2
Age, median (min-max)	46(22–55)	42(19–59)	35(20–58)	42(20–60)	40(20–58)
Education, years ± SD	15.1 ± 2.2	16.1 ± 1.1	15.1 ± 2.4	15.2 ± 2.4	16.4 ± 1.8

Data are presented as mean ± SD. Significant differences relative to the Control group: *—*p* < 0.05 (chi-square criteria).

## Data Availability

Data are unavailable due to privacy or ethical restrictions.

## Supplementary Materials

The following supporting information can be downloaded at https://www.mdpi.com/article/10.3390/ijms262412141/s1.
